# Dual indexed library design enables compatibility of in-Drop single-cell RNA-sequencing with exAMP chemistry sequencing platforms

**DOI:** 10.1186/s12864-020-06843-0

**Published:** 2020-07-02

**Authors:** Austin N. Southard-Smith, Alan J. Simmons, Bob Chen, Angela L. Jones, Marisol A. Ramirez Solano, Paige N. Vega, Cherie’ R. Scurrah, Yue Zhao, Michael J. Brenan, Jiekun Xuan, Martha J. Shrubsole, Ely B. Porter, Xi Chen, Colin J. H. Brenan, Qi Liu, Lauren N. M. Quigley, Ken S. Lau

**Affiliations:** 1 Epithelial Biology Center and Department of Cell and Developmental Biology, Vanderbilt University School of Medicine, Nashville, TN USA; 2 Chemical and Physical Biology Program, Vanderbilt University, Nashville, TN USA; 3 Vanderbilt Technologies for Advanced Genomics, Vanderbilt University Medical Center, Nashville, TN USA; 4 Center for Quantitative Sciences, Vanderbilt University Medical Center, Nashville, TN USA; 5 RootPath Genomics, Inc., Cambridge, MA USA; 6 1CellBio, Inc., Watertown, MA USA; 7 Vanderbilt Ingram Cancer Center, Nashville, TN USA; 8 Department of Medicine, Division of Epidemiology, Vanderbilt Epidemiology Center, Vanderbilt University Medical Center, Nashville, TN USA

**Keywords:** Single-cell RNA sequencing, inDrop, TruSeq, Next-generation sequencing, NovaSeq, Index hopping, Multiplexing, Exclusion amplification

## Abstract

**Background:**

The increasing demand of single-cell RNA-sequencing (scRNA-seq) experiments, such as the number of experiments and cells queried per experiment, necessitates higher sequencing depth coupled to high data quality. New high-throughput sequencers, such as the Illumina NovaSeq 6000, enables this demand to be filled in a cost-effective manner. However, current scRNA-seq library designs present compatibility challenges with newer sequencing technologies, such as index-hopping, and their ability to generate high quality data has yet to be systematically evaluated.

**Results:**

Here, we engineered a dual-indexed library structure, called TruDrop, on top of the inDrop scRNA-seq platform to solve these compatibility challenges, such that TruDrop libraries and standard Illumina libraries can be sequenced alongside each other on the NovaSeq. On scRNA-seq libraries, we implemented a previously-documented countermeasure to the well-described problem of index-hopping, demonstrated significant improvements in base-calling accuracy on the NovaSeq, and provided an example of multiplexing twenty-four scRNA-seq libraries simultaneously. We showed favorable comparisons in transcriptional diversity of TruDrop compared with prior inDrop libraries.

**Conclusions:**

Our approach enables cost-effective, high throughput generation of sequencing data with high quality, which should enable more routine use of scRNA-seq technologies.

## Background

Most droplet-based single-cell RNA-seq (scRNA-seq) libraries to date have been sequenced on Illumina sequencing platforms using their sequencing-by-synthesis technology [1–3]. Libraries generated by droplet-based scRNA-seq approaches require a certain read depth for adequate identification of cell types and states [1, 2]. With the introduction of Illumina’s NovaSeq6000 next generation sequencing (NGS) platform, the number of scRNA-seq libraries that can theoretically be multiplexed for sequencing together to the required depth has significantly increased [4]. Coupled with improvements in hardware technology and sequencing chemistry, sequencing costs can be dramatically reduced, which in turn can facilitate scRNA-seq for routine laboratory use (Supplementary Table 1). However, the utilization of the improved exclusion amplification (ExAmp) chemistry and patterned flow cells in this new technology has introduced new problems for droplet-based scRNA-seq library structures to date [5–9].

**Fig. 1:**
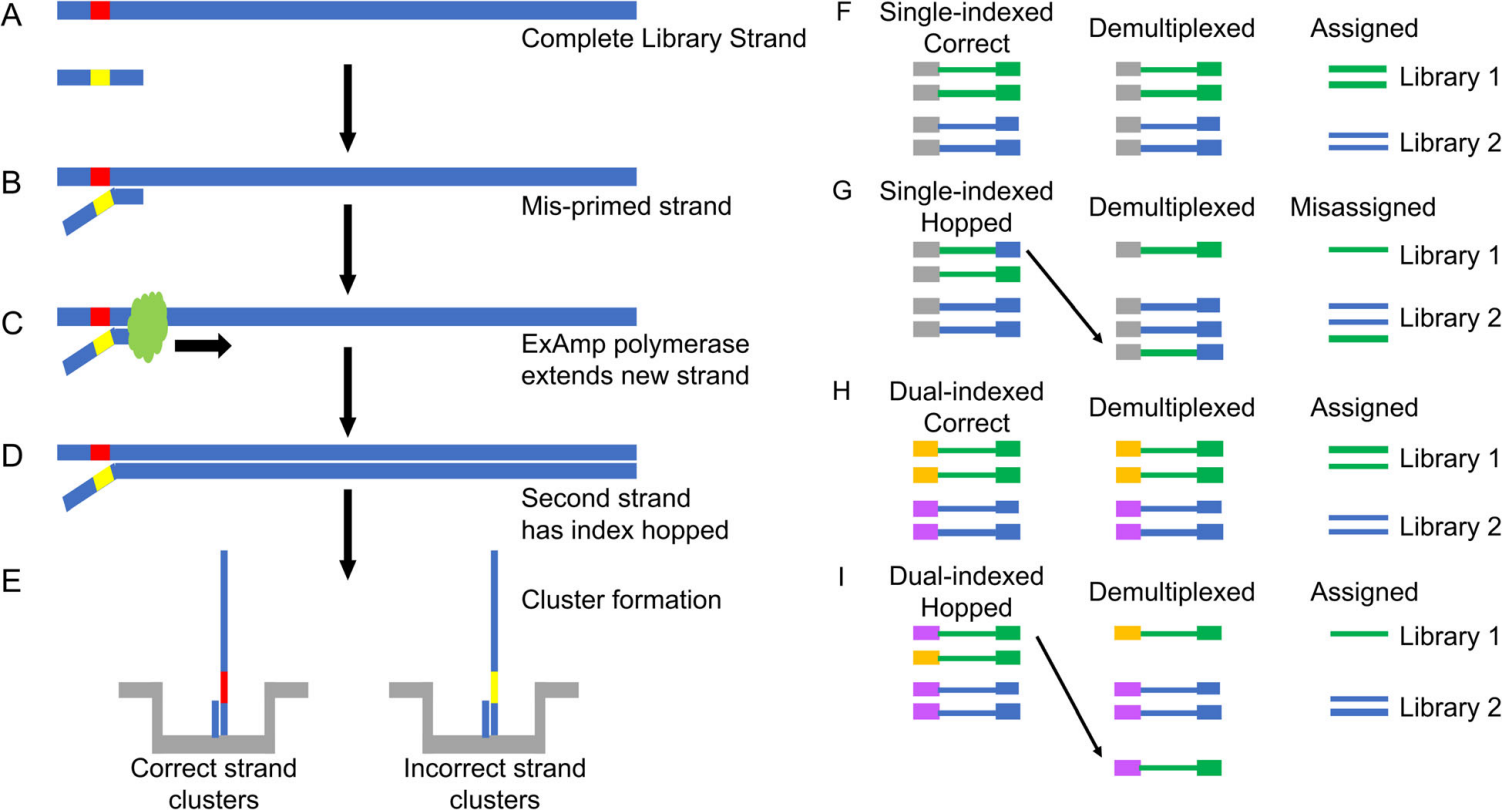
Mechanism for index hopping and its effects on sequencing library demultiplexing. **a**-**e** Illustration of index hopping due to (**a**) free adapter molecules remaining after purification post-PCR, resulting in (**b**) mis-priming of a single stranded library molecule. **c** The mis-primed library molecule is extended via ExAmp polymerase to generate (**d**) a fully complete library molecule with an incorrect sample index assigned. **e** Both correct and index-hopped molecule can form clusters on the flow cell. **f**-**i** Demultiplexing runs with single- or dual-indexed libraries with index hopping. **f** The case with a single index and no index hopping where the read(s) for a cluster are associated with a specific sample index (green with green and blue with blue) added to each molecule during library preparation, allowing reads to be assigned to its correct library of origin. **g** The case as above but with index hopping (a blue index now marks a green cluster), where that read will be incorrectly assigned to the wrong library. **h** A unique dual-indexed strategy allows for a single sample to have 2 indexes to be associated with a single library molecule. Here, library 1 = yellow + green, library 2 = purple and blue. **i** The case as above but with index hopping will result in reads displaying unanticipated combination of indexes (e.g., purple + green). The reads associated with unanticipated indexes can then be filtered out

One aspect to be considered when sequencing using ExAmp chemistry is the increased rate of index-hopping between samples sequenced together compared with those sequenced using Illumina’s normal bridge amplification chemistry [6]. It has been previously documented that index hopping occurs due to the physical incorporation of the sample index from one library into a library molecule from a different library (Fig. 1a-e) [7, 8]. The end result is the mis-assignment of reads between samples (Fig. 1f-i). Index hopping presents a significant problem for scRNA-seq libraries, where data resolution and sample integrity are vitally important. While computational approaches to use cell barcodes as a second index to solve this mis-assignment problem have been proposed [8, 9], due to the redundant nature of barcodes used in different bead lots, a large amount of data will need to be discarded due to cross-sample barcode collisions. Depending on the number of libraries sequenced, this can be well over 20%. Kircher, M et al. previously demonstrated that individual index-hopped reads can be filtered out of the final data by incorporating a second sample index (i5) on the other side of the final sequencing library (Fig. 1h-i) [10]. Using this established solution, an index-hopped read would be identified by an un-anticipated combination of sample indexes and can be filtered out. Currently, using a second index and proper sample handling to prevent sample mixing prior to sequencing are the only methods available to pro-actively prevent index-hopping in bulk sequencing assays [7, 10].

There are several issues to consider when designing a dual-indexed scRNA-seq library that is compatible with the NovaSeq. A combinatorial dual-indexing scheme in which at least one of the two sample indexes is repeated across two or more samples will reduce the samples that could be potentially mis-assigned. However, samples sharing a sample index would still need to be treated as a single-indexed library (Fig. 1g) [6]. The best method then is to use a unique dual-indexed system (Fig. 1i) so that none of the sample indexes on one side of the library (i7) or the other (i5) are shared between samples [6]. The indexes used for both sides of the library should be sufficiently different that a single base error (insertion, deletion, or substitution) should not result in the mis-assignment of the associated read [11].

For the original inDrop V2 method, a high-throughput, droplet-based microfluidic scRNA-seq method, the single sample index is added on at the very end of library preparation. Initially, a cell is co-encapsulated with a hydrogel bead coated in poly T capture oligonucleotides also containing barcodes unique to each bead, and hence cell, partial R1 sequencing primer sites, and a T7 promoter. The transcripts from each cell are captured and reverse transcribed (RT) to DNA before being converted to double-stranded DNA (dsDNA) in a second strand synthesis reaction. The library is then linearly amplified by an in-vitro transcription step using the T7 promoter before being converted back to cDNA during an RT and subsequent PCR reaction. These final two steps (RT and PCR) are where the custom sequencing priming sites and sample index are added and completed in the V2 structure. These custom sequencing primers from the prior inDrop V2 library structure are incompatible with other Illumina libraries, such as common TruSeq libraries. They can mis-prime Illumina libraries and vice versa, resulting in loss of inDrop sequence data when V2 libraries are sequenced in multiplexed library pools where the majority of libraries are Illumina libraries [2, 12]. Thus, previous sequencing runs of V2 scRNA-seq libraries occupy the entire sequencing flow cell (Methods). When sequencing just a single library type, the resulting low base composition diversity during the spacer region of the inDrop V2 cell barcode read results in a spike in base call error rate. The ability to sequence alongside other Illumina libraries should increase the diversity of bases incorporated across the flow cell at each cycle, improving not only the base calling accuracy, but also the flow cell cluster recognition during sequencing [13]. Prior work in improving the inDrop library involved changing the RNA capture oligonucleotide sequences, restricting the solution to only those who could generate custom inDrop capture beads in-house [14, 15]. Other alterations in library structure has not been thoroughly tested for compatibility nor library quality in the new generation of sequencers such as the NovaSeq6000 [14].

Here, we document the development and benchmarking of an Illumina compatible dual-indexed library structure for the inDrop scRNA-seq platform that builds upon the widely-used, commercially available V2 gel beads in a manner independent of the cell barcodes incorporated into the library. We demonstrate how transitioning to a uniquely dual-indexed library with standard sequencing primers allows for greater sequencing throughput and quality of inDrop scRNA-seq. Using the design documented here, anywhere from 1 to 96 of the resulting scRNA-seq libraries can be sequenced alongside other Illumina samples with minimal sample cross-talk, as well as improvements in sequencing accuracy, which should facilitate the widespread adoption of scRNA-seq in experimental workflows.

## Results

### Sequencing quality of inDrop scRNA-seq libraries is improved when sequenced with a diverse Illumina library

Previously, it was unknown if certain features of inDrop libraries, such as the cell barcodes and spacer region, would interfere with the performance of other Illumina libraries (and vice versa) during sequencing. To assess compatibility with Illumina TruSeq libraries, inDrop V2 libraries were sequenced alongside a 10–15% spike in of Illumina’s PhiX control library, compared to a run without PhiX. Sequencing on both a low-throughput nano run on MiSeq, as well as a mid-throughput NextSeq run, were successful with appreciable number of reads from inDrop V2 libraries (87.8 and 110.9% of the target read depth, respectively; Table 1).

**Table 1:** Sequencing yield and quality of V2 inDrop with/without standard illumina libraries

Sequencing Run	Sequencer	Sequencing Kit	Targeted inDrop read depth	Observed inDrop read depth	Mean transcript Quality Score	Mean Barcodes and UMI Quality
V2 structure mouse 1	NextSeq	Mid-throughput	130,000,000	148,238,920	30.72	30.55
V2 structure mouse 1 + 10% illumina PhiX	MiSeq^a^	Nano	900,000	745,903	34.94	32.24
V2 structure mouse 2 and 3 + 15% illumina PhiX	NextSeq	Mid-throughput	110,500,000^b^	122,520,660	33.09	33.08

^a^It is thought that the inDrop reads (745,903) for the MiSeq test was lower than the expected 1 million reads due to the fact that the loading concentration of inDrop libraries has been optimized on the NextSeq, but not on the MiSeq. On the NextSeq we have found that loading the inDrop libraries at 1.5x the listed optimal loading concentration improves clustering efficiency on the flow cell. The loading concentration of inDrop libraries on the MiSeq for this sequencing run was just the standard loading concentration

^b^The targeted read depth is slightly decreased here compared to that of the V2 Structure mouse 1 because 15% of the read depth is expected to be taken up by PhiX

**Fig. 2:**
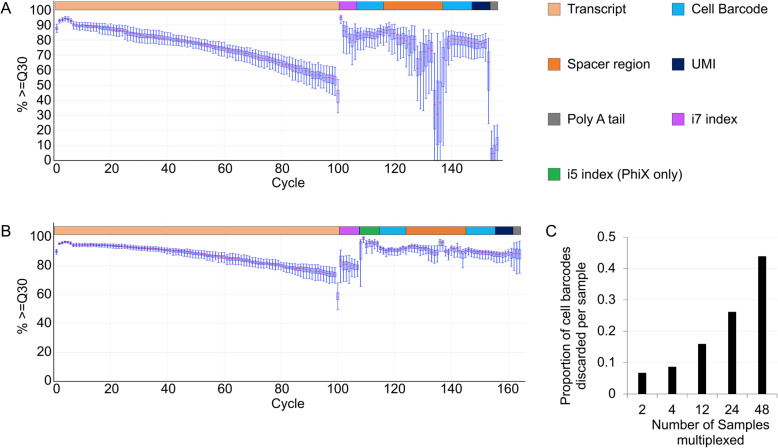
Quality of single-indexed inDrop libraries sequenced alongside Illumina libraries and data loss from index hopping. **a** The base calling accuracy plot for a inDrop V2 library on a NextSeq sequencing run, depicting the spread of quality scores as each base is sequenced. This plot consists of a series of box plots where each box plot maps the percent of clusters in each image of the flow cell with quality scores ≥30 (called Q30) in each cycle. The first 100 cycles correspond to the transcript read; the next 6 correspond to the i7 index read; the final 50 correspond to the cell barcode + UMI reads. The last 6 cycles read into the poly A tail due to the variable length of the inDrop cell barcodes. **b** The base calling accuracy plot for a inDrop V2 library sequenced alongside the control Illumina library, PhiX, on a NextSeq. When sequencing alongside PhiX, the 7-base long i7- and i5- index reads are used so that PhiX reads can be filtered out and discarded during demultiplexing. **c** Plot of the calculated proportion of cell barcodes that need to be discarded from single-indexed sequencing runs at different levels of multiplexing. We assume each sample will contain ~ 3000 cell barcodes

Importantly, sequencing inDrop libraries with PhiX resulted in mean quality score increases for both the transcript read and the barcode + UMI (unique molecular identifier) read (Table 1) [16]. The improved quality scores equate to a decrease in the probability of an error in base calling from 8.803 × 10^−4^ to 4.917 × 10^−4^ on the transcript read, and a corresponding decrease in error probability from 8.455 × 10^−4^ to 4.908 × 10^−4^ on the barcode + UMI read. This represents about a 1.8- and 1.7-fold decrease in the base calling error rate for bases incorporated during sequencing. This is also reflected in the base calling accuracy plots from the two sequencing runs (Fig. 2a-b). The base calling accuracy plot describes the spread of quality scores as each base is sequenced. It is interpreted as a series of box plots where each box plot maps the percent of clusters in each image of the flow cell with quality scores ≥30 (referred to here as Q30) in each flow cell imaging cycle. When inDrop V2 and Illumina PhiX were sequenced together (Fig. 2b), the transcript read (cycles 1–100) median Q30 barely droppedbelow 80% from cycles 80–100, whereas the inDrop V2 only library median Q30 decreased below 60% during cycles 80–100 (Fig. 2a). In addition, for combined libraries, the Q30 scores during the barcode + UMI read (cycles 114–164) were maintained at or above 80% for most of the cell barcode + UMI read (Fig. 2b). These results demonstrate that inDrop V2 libraries are compatible with low concentrations of standard Illumina libraries for sequencing, and that when sequenced together, the sequencing quality, especially for the non-diverse barcode region, is improved for inDrop libraries. The decreases in targeted read depth and observed sequencing reads (122 million vs 148 million, Table 2) when sequencing V2 libraries alongside PhiX resulted from PhiX utilizing some of the total available read depth on the flow cell. Because both inDrop sequencing runs on the NextSeq over-clustered to a similar degree here (10% with PhiX and 14% without PhiX) (Table 1), this factor was thought to be inconsequential to the observed quality scores. The increases in quality scores were likely due to sequencing alongside the PhiX control library, which has a high diversity of bases represented at each position of the sequencing library. This would result in easier cluster recognition on the flow cell [13].

**Table 2:** Evaluation of the raw yield and quality of TruDrop libraries when sequenced on the NovaSeq

Library	Sequencer	i7	i5	Targeted inDrop Read Depth	Observed inDrop Reads	Average % of the lane	Percent perfect index reads	Mean transcript Quality Score	Mean Barcodes and UMI quality score
TruDrop Mouse 4	NovaSeq 6000	CCGCGGTT	AGCGCTAG	50,000,000	53,655,662	0.64%	96.99%	35.57	36.22
TruDrop Mouse 5	NovaSeq 6000	TTATAACC	GATATCGA	50,000,000	44,554,464	0.53%	94.13%	35.53	36.19
V2 Mouse 2 + 15% illumina PhiX	NextSeq	GATATCGA	–	65,000,000	57,847,546	37.68%	91.72%	33.06	33.02
V2 Mouse 3 + 15% illumina PhiX	NextSeq	GCCAAT	–	65,000,000	64,673,114	42.14%	92.64%	33.12	33.14
V2 Mouse 4 + 99% Illumina PhiX	NovaSeq 6000	CTTGTA	–	50,000,000	10,985,817	0.09%	–	–	–
V2 Mouse 5 + 99% Illumina PhiX	NovaSeq 6000	GTGAAA	–	50,000,000	10,144,573	0.08%	–	–	–
V2 Mouse 4 + 0% Illumina PhiX	NextSeq	CTTGTA	–	100,000,000	97,872,275	24%	–	31.89	28.67
V2 Mouse 5 + 0% Illumina PhiX	NextSeq	GTGAAA	–	100,000,000	92,742,820	23%	–	32.03	28.09

### Redesigned inDrop library structure potentially enables higher-throughput NGS

Having demonstrated the compatibility of inDrop V2 library features with standard Illumina libraries in NGS, we next sought to re-engineer the inDrop library structure for higher-throughput, ExAmp chemistry-based sequencers, such as the NovaSeq6000. Specifically, we sought to incorporate dual-indexing to overcome the well-documented index hopping problem on the NovaSeq [5]. If two single-indexed samples share cell barcodes and index hopping occurs, then it will be impossible to determine the origins of a particular read belonging to the shared barcode, resulting in the discarding of cells with shared barcodes across indices. We call this problem cross-sample barcode collision, and calculated the theoretical amount of data discarded upon multiplexed NovaSeq runs (Supplementary File 1). For pools of 2, 4, 12, 24, and 48 samples the percentages of cell barcodes, and hence cells, discarded due to cross sample barcode collisions are 8.67, 15.99, 26.19, and 43.87%, respectively (Fig. 2c) [1, 2, 17, 18].

**Fig. 3:**
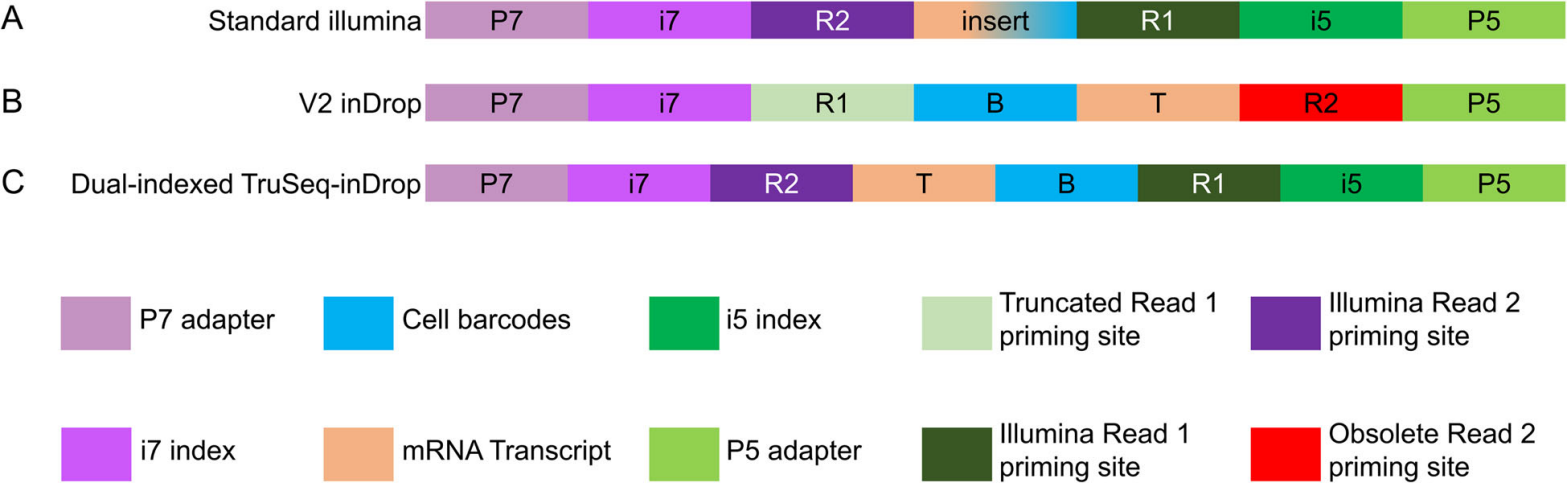
Variations of inDrop library structures from the perspective of sequencing. **a** A standard Illumina library contains P7 and P5 adapter sites that are used to bind Illumina sequencing flow cells. i7-and i5-indexes are incorporated onto the P7 and P5 sides, respectively, to adopt a dual-indexing strategy. On either side of the insert are sites (R1 and R2) where standard Illumina sequencing primers are used to read across both sides of the insert. The reverse complement of these read priming sites then allows for the priming and subsequent reading of the i7 and i5 sample indexes. **b** The inDrop V2 library structure also incorporates the P7 and P5 flow cell adapter binding sites, with a single i7 index. The V2 structure utilizes a R1 priming site that is a truncated version of the standard R2 priming site, and a R2 priming site that is a deprecated R2 priming site. In addition, the R1 and R2 of the V2 structure are flipped so that the insert is read backwards from a normal Illumina library. **c** The TruSeq-inDrop (TruDrop) structure incorporates a second (i5) index and the standard Illumina R1 and R2 priming sites that are used in all Illumina TruSeq libraries

To minimize the possibility of cross-sample barcode collision, a second i5 index was incorporated when designing the new library structure. The i5 and i7 indexes used follow a unique-dual indexing strategy such that when only considering one side of the library, each index is only used once. During the redesigning process, it was discovered that the i7 index custom sequencing primer for the V2 library structure shares a 13 bp region on the 5′ end with the standard Illumina sequencing primer. This region is built into the oligonucleotide used for the barcoded inDrop hydrogel capture beads [2, 12]. Thus, when sequencing alongside standard Illumina libraries that make up the majority of the library and primer pools, it is expected that a large portion of V2 library strands will mis-prime during the i7 index read with standard Illumina sequencing primers, resulting in poor identification of i7 indexes for clusters on the flow cell. The degree of mis-priming is a function of the reaction kinetics driven by the relative concentrations of the incompatible primers. inDrop clusters that can be properly identified during the index read will also be lower quality. Due to this incompatibility of the i7 sequencing primer, it was thus decided that the newer libraries would use the dual indexed, Illumina TruSeq library Structure. The incorporation of the standard Illumina sequencing primer binding sites allows for sequencing of TruDrop libraries in sequencing pools with other Illumina libraries as currently performed on NovaSeq (Table 2). The new library incorporates standard Illumina TruSeq adapter sequences [12], the P5 and P7 flow cell binding sites, the TruSeq standard sequencing primer binding sites (in contrast to prior V2 libraries which require custom sequencing primers), and unique dual indexes (Fig. 3). Furthermore, to achieve a standard Illumina TruSeq library structure, the cell barcode + UMI read was swapped to read 1, which has previously been documented as the higher quality read [19]. Since these indexes are designed to be pooled in sets of 8 index pairs [20] and the maximum number of libraries that can be sequenced to a read depth of ~ 100 million reads per sample on a single NovaSeq lane is 25 [4], we selected 24 index pairs (24 unique “i7” and 24 unique “i5”) to be used as the new indexes in the new library structure. Theoretically, the number of usable index pairs can be increased to 3840 using IDT’s set of 10 bp unique dual indexes, although they have to be individually validated. We call this new library structure TruSeq-inDrop (TruDrop). The modifications required for TruDrop library preparation rely on the substitution of primer sequences for those of their V2 counterparts (Methods), without requiring the engineering of new beads nor design of a new library preparation protocol. This change maximizes accessibility to the current users of inDrop. The final sequence for the barcode + UMI and transcript sides of TruDrop libraries are as follows:

Cell Barcodes: 5′ – AATGATACGGCGACCACCGAGATCTACAC [i5] ACACTCTTTCCCTACACGACGCTCTTCCGATCT [cell barcode 1] GAGTGATTGCTTGTGACGCCTT [cell barcode 2][UMI]TTTTTTTTTTTTTTTTTTT … – 3′. Transcript: 5′ – CAAGCAGAAGACGGCATACGAGAT [i7]GTGACTGGAGTTCAGACGTGTGCTCTTCCGATCTNNNNNN … – 3′.

A detailed version of the custom primers and indexes for library preparation of TruDrop libraries can be found in the supplementary materials (Supplementary Files 2 and 3).

### TruDrop primers function similarly to V2 primers during inDrop library preparation

As TruDrop uses redesigned primers to generate libraries compatible with TruSeq libraries, it is important to verify that all indexes can be appropriately used to complete and amplify inDrop libraries during the final stages of library preparation. Of the initial 24 tested, all but one (TruDrop index pair 9) yielded qPCR amplification curves similar to those of V2 primer pairs (Supplementary Fig. 1A). Furthermore, the Ct values of TruDrop primer pairs 1–8 and 10–24 were well within 1.5 cycles of the average Ct (Supplementary Fig. 1B), suggesting little to no difference in amplification bias between the new primers and the prior V2 primers. As TruDrop index pair 9 failed to amplify appropriately when compared to V2 primers, it was replaced with index pair 25 (which behaved similar to V2 primers) in all further testing.

### TruDrop libraries see improved performance when sequenced using exAMP chemistry

**Fig. 4:**
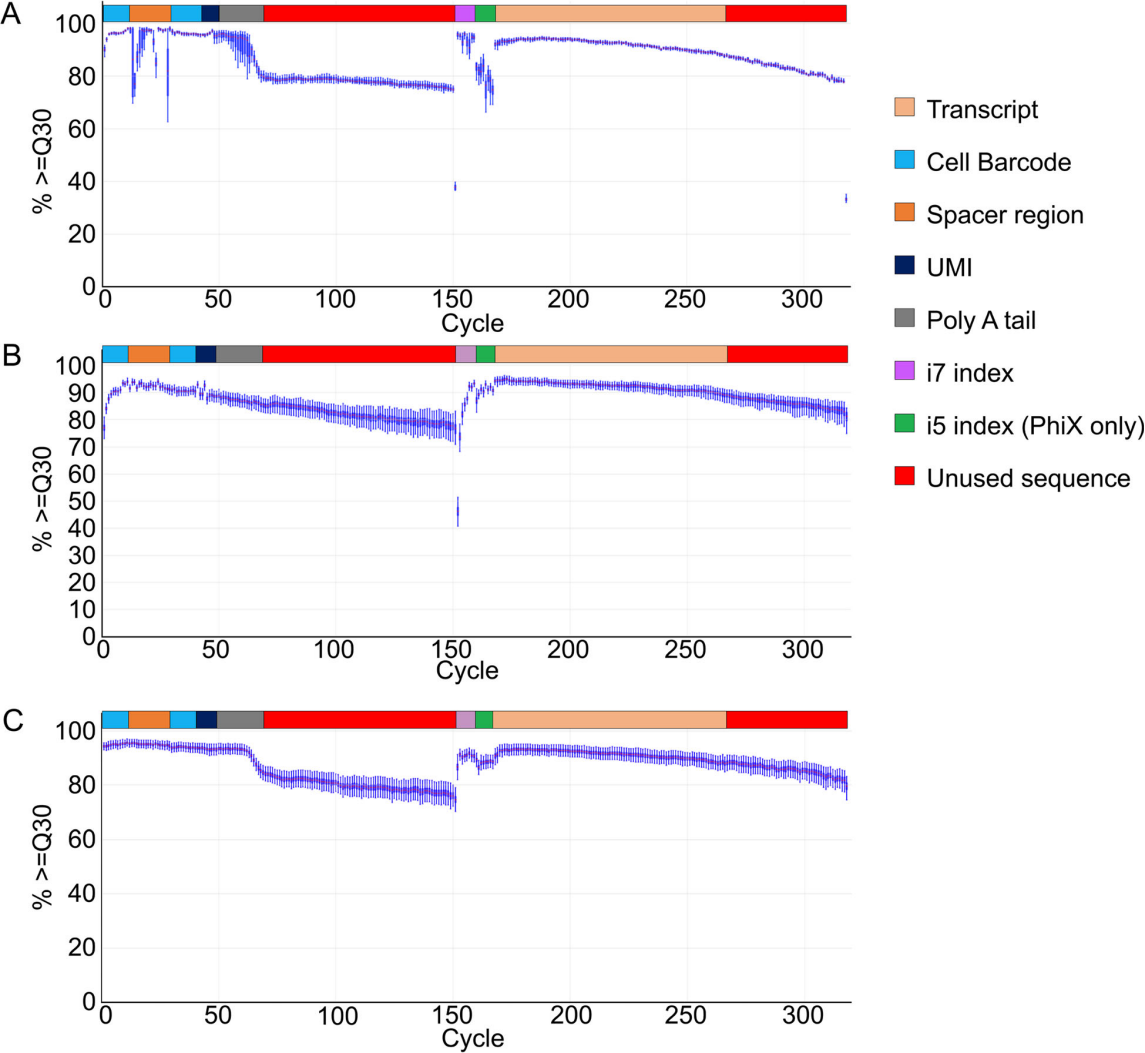
Sequencing quality of TruDrop libraries on exAmp chemistry sequencers. **a** The base calling accuracy plot for two dual-indexed TruDrop libraries on iSeq alongside PhiX. Cycles 1–50 depict the quality scores for the cell barcode + UMI read. Cycles 51–151 are sequence data that will be trimmed and discarded during analysis. Cycles 152–159 correspond to the i7 index read. Cycles 160–167 are the i5 index read. Cycles 168–318 are the transcript read. For the purpose of direct comparison only cycles 168–267 are marked as transcript as only 100 bases of transcript were sequenced for the V2 libraries. **b** The base calling accuracy plot for the same two TruDrop libraries when sequenced on the NovaSeq alongside 107 other libraries. **c** The base calling accuracy plot for 24 dual-indexed TruDrop library sequenced on a NovaSeq alongside 186 other libraries

To put TruDrop libraries into action, we first sequenced these libraries on the iSeq 100, which utilizes patterned flow cells and ExAmp chemistry to test clustering efficiency and priming effectiveness during the sequencing run [21, 22]. Two V2 libraries that had previously performed well on the NextSeq (yielding 97.9 and 92.7% of the target 100 million read depth per library on the NextSeq) were prepared from the same starting material as TruDrop libraries (Table 2). The TruDrop samples were then sequenced alongside PhiX on the iSeq 100, yielding an average of 151% of the 2 million reads per library target read depth (Supplementary Table 2). The median Q30 remained at or above 90% during most of the barcode + UMI cycles (cycles 1–11 and 31–50). While for the transcript cycles (cycles 167–316), the median Q30 remained at or above 80% for the full 150 cycle transcript read (Fig. 4a). However, if only the first 100 bases of the transcript read (the same length as the NextSeq read length) were considered, then 90% or more of reads were above Q30. Thus, it was expected that TruDrop libraries can be sequenced on the NovaSeq but also see improved read quality scores compared to V2 libraries sequenced on the NextSeq with PhiX.

The same TruDrop libraries were then sequenced on the NovaSeq6000 alongside 107 other standard Illumina libraries (Table 2). The TruDrop libraries yielded 107 and 89.1%, respectively, of their target read depth (50 million reads per library), accounting for 0.64 and 0.53%, respectively, of the three NovaSeq lanes they were on. Since these TruDrop libraries were sequenced alongside a large number of other standard Illumina libraries, the overall base composition of the libraries was very diverse and corresponded to sequencing alongside PhiX. Compared to prior tests with V2 libraries on the NextSeq, this was the equivalent of sequencing alongside 99% PhiX (due to the increased diversity of base composition associated with sequencing alongside many library types) with no loss in targeted read depth. In addition, there was an increase of 1.5–5.3% in the number of flow cell clusters with perfect index reads compared to V2 libraries on the NextSeq (Table 2). Quality scores were further improved, corresponding to a 2.1- and 1.8-fold reduction in base call error rate compared with sequencing V2 libraries on the NextSeq with PhiX, and a 3.7- and 3.0-fold decrease compared to sequencing just V2 libraries alone on the NextSeq. The base call accuracy plot reflects this improvement (Fig. 4b), as 90% or more of reads from TruDrop libraries during read 1 (cell barcode + UMI) and read 2 (transcript) that are of interest in inDrop libraries are at or above Q30. These results demonstrate that not only can TruDrop libraries be sequenced on the NovaSeq, they also see significant improvements in the sequencing quality for both the transcript and barcode + UMI regions.

To provide a direct comparison of the performance of TruDrop libraries with inDrop V2 libraries under the same condition, the V2 libraries (Table 2) for the corresponding TruDrop samples were also sequenced on a single NovaSeq sequencing run targeting the same read depth of 50 million reads per library. Compared to the TruDrop yields of 107% and 89.1% of target read depth, the V2 libraries yielded only 22.0 and 20.3% of the target read depth on the NovaSeq, as compared to 97.9 and 92.7% when these V2 libraries were sequenced on the NextSeq. These results validate our case that V2 libraries will perform poorly on a shared NovaSeq run due to mis-priming of both inDrop and Illumina clusters on the flow cell. Thus, we demonstrate that to utilize the NovaSeq for sequencing inDrop libraries properly, the TruDrop library structure should be used.

### TruDrop libraries maintain high quality when multiplexed in a high throughput fashion

With the successful testing of the two initial pairs of indices on the NovaSeq, 24 human and mouse samples were prepared and sequenced, each uniquely dual-indexed, on the NovaSeq6000 alongside 186 other Illumina libraries. There was no observed change in the distribution of library size profiles (Supplementary Fig. 2). TruDrop libraries yielded 94–151% of the target 125 million reads per sample (Supplementary Table 3). In total, the 24 samples represented 29.4% of the raw sequencing yield across all of the lanes from the flow cell. This was equivalent to sequencing alongside ~ 70% PhiX, as compared to the previous run with ~ 99% PhiX equivalents. However, the quality scores and error rates were observed to be maintained even with a decrease percentage of diverse libraries due to the large majority of diverse libraries still present. The average transcript and barcodes + UMI quality scores were 35.32 and 36.07, respectively, (Supplementary Table 3). These do not differ greatly from the prior TruDrop NovaSeq sequencing run (Table 2) and are still a 2.0- and 1.7- fold reduction in base call error rate over V2 libraries on the NextSeq with PhiX, and a 3.6- and 2.9-fold reduction in error over just V2 libraries alone on the NextSeq. These results suggest that the improved quality scores observed on the NovaSeq can be maintained as long as some minimum diversity of Illumina libraries are present.

Given the apparent improvement in the quality scores (and associated decrease in error rates) of the TruDrop libraries on the NovaSeq, we compared the data from the 26 libraries we had sequenced so far to 11 inDrop V2 samples that we had previously sequenced on the NextSeq (Supplementary Table 4). A Mann-Whitney test indicated that the distribution of transcript quality scores for TruDrop libraries on the NovaSeq (Median = 35.36) were statistically different (*P* = 2.339 × 10^-9) from that of V2 libraries on the NextSeq (Median = 32.03) (U = 0, n_TruDrop_ = 26, n_V2_ = 11, two tailed). A second Mann-Whitney test indicated that the distribution of barcodes + UMI quality scores for TruDrop libraries on the NovaSeq (Median = 36.09) were statistically different (P = 2.339 × 10^-9) from that of V2 libraries on the NextSeq (Median = 29.78) (U = 0, n_TruDrop_ = 26, n_V2_ = 11, two tailed).

The base calling accuracy plot also depicts this improvement in base calling accuracy, as the region covering the cell barcodes + UMI (cycles 1–11 and 31–50) displays more than 90% of the reads above Q30 (Fig. 4c). For the first 100 transcript read bases, 90% or more of the reads were at or above Q30. The drop observed in the base calling accuracy plot at cycle 60 that continues to the end of read 1 (cycle 150) corresponds to the location of the poly T capture sequence. This decrease in accuracy only continued through regions that would be trimmed out during mapping and barcode deconvolution. The decrease in accuracy did not affect other Illumina libraries on the flow cell, as when considered individually, 95% of other Illumina libraries had greater than 90% of reads at or above Q30 for the entire sequencing run. These results demonstrate that up to 24 TruDrop libraries can be multiplexed on the NovaSeq alongside standard Illumina libraries, while maintaining a very high sequencing quality for both inDrop and Illumina libraries. With lane splitting, 4 pools of 24 samples can be sequenced across 4 sequencing lanes for a total of 96 inDrop libraries sequenced at a time.

### TruDrop libraries on the NovaSeq have improved sequence alignment rates

To investigate if the improvement in base call accuracy had a measurable effect on downstream data quality, two colonic (one mouse and one human) libraries that had previously been sequenced as V2 libraries on the NextSeq were re-made with the TruDrop structure and sequenced on the NovaSeq. Both mouse and human libraries were tested to ensure general applicability. The reads for the sequenced V2 libraries and the TruDrop libraries were then aligned and deconvolved in parallel. The overall percentage of reads that aligned did not significantly change from V2 to TruDrop libraries for either the mouse (96.38 and 96.56%, respectively) or human (96.11 and 95.12%, respectively) replicates (Table 3). However, for the mouse sample, the percentage of unique alignments increased from 67.15 to 73.48%, while the human sample experienced a similar improvement from 84.44 to 87.23%.

**Table 3:** Comparison of data alignment quality of the inDrop V2 and TruDrop structures

Sample	Sequencer	Sequencing Depth (reads)	mapped reads (%)	Uniquely aligned reads (%)
V2 Mouse	NextSeq	98,606,967	96.38	67.15
TruDrop Mouse	NovaSeq	43,657,381	96.56	73.48
V2 Human	NextSeq	55,507,773	96.11	84.44
TruDrop Human	NovaSeq	188,061,057	95.12	87.23

To further quantify the improvement in sequence quality, we compared the alignment and barcode deconvolution efficiency of 21 TruDrop libraries to that of 11 inDrop V2 libraries (Supplementary Fig. 3, Supplementary Table 5). A Mann-Whitney test indicated that the percent of reads containing valid barcodes in TruDrop libraries sequenced on the NovaSeq (Median = 97.19%) was significantly higher (*P* = 3.023 × 10^-6) than in the V2 libraries on the NextSeq (Median = 88.46%) (U = 11, n_TruDrop_ = 21, n_V2_ = 11, two-tailed). Similarly, the percent of reads uniquely aligning to the reference genome in TruDrop libraries sequenced on the NovaSeq (Median = 79.45%) was also significantly higher (*P* = 7.143 × 10^-3) than in V2 libraries sequenced on the NextSeq (Median = 74.63%) (U = 49, n_TruDrop_ = 21, n_V2_ = 11, two-tailed). Consequently, the percentage reads in a library containing a valid cell barcode and uniquely aligning to the reference genome in TruDrop libraries sequenced on the NovaSeq (Median = 64.09%) was significantly higher (*P* = 1.243 × 10^-4) than in V2 libraries on the NextSeq (Median = 56.06%) (U = 25, n_TruDrop_ = 21, n_V2_ = 11, two-tailed). Thus, improved data quality can be observed consistently for TruDrop libraries sequenced on the NovaSeq.

### Single-cell data generated by TruDrop maintain the same cell population structure as inDrop V2

To assess the validity of TruDrop libraries at the single-cell transcriptomic level, cell-by-gene count tables were generated by the same alignment, deconvolution, and filtering procedures used for inDrop V2 libraries. We determined that the numbers of UMIs recovered from TruDrop libraries were comparable to those of inDrop V2 libraries routinely generated for colonic tissues at similar read depths. With TruDrop libraries sequenced on NovaSeq, we observed medians of ~ 11,500 UMI/cell and 3208 genes/cell when libraries were sequenced to ~ 60 K reads per cell, which were in the same order of magnitude of ~ 6000 UMI/cell in data previously generated using inDrop V2 and NextSeq (Supplementary Table 6) [23, 24]. When increasing the sequencing depth to ~ 150 K reads per cell, TruDrop libraries yielded an output of ~ 16,000 UMI/cell and ~ 3800 genes/cell.

**Fig. 5:**
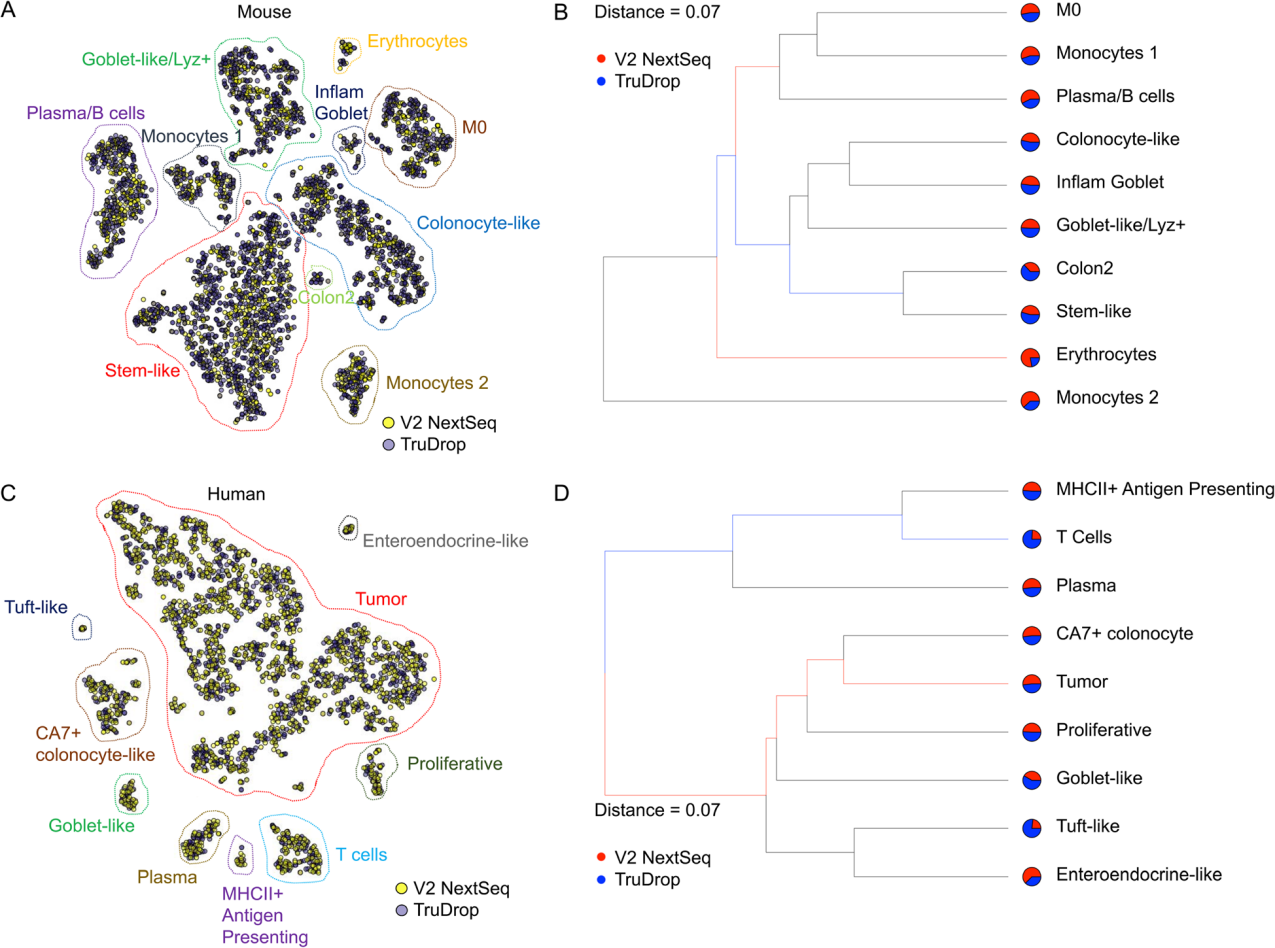
Comparison of cell types identified between inDrop V2 libraries on NextSeq and TruDrop on NovaSeq. **a** and **c** Combined t-SNE analysis of cells identified from a TruDrop and V2 library prepared from the same samples of (**a**) mouse and (**c**) human tumors. **b** and **d** sc-UniFrac tree representations of subpopulation structures for libraries presented in **a** and **c**, respectively. Cell groups enriched using V2 NextSeq libraries have red branches, while those enriched using TruDrop NovaSeq have blue branches. Thickness of branches represent level of enrichment. Distance values range from 0 to 1, with 0 representing complete overlap between two datasets

To directly compare cell populations detected by the two library preparation procedures, data generated by TruDrop and inDrop V2 from technical replicate samples were co-embedded using t-SNE [25]. Analyses using sets of both human and mouse colonic tumor samples revealed significant intermixing between TruDrop and V2, with identical cell types detected (Fig. 5a, c, Supplementary Fig. 4A). To quantify this mixing, we used sc-UniFrac [23], a distance metric between 0 and 1, with 0 signifying two samples to be identical and 1 signifying complete non-overlap. For both mouse and human sample sets, the sc-UniFrac distance was 0.07, strongly suggesting that cell populations identified with the different libraries were almost completely identical (Fig. 5b, d Supplementary Fig. 4B), with minor differences (such as in erythrocytes) due to the small number of cells in those clusters. These data suggest that the library structure and sequencer used did not result in any overt biases in data for recovering cell types.

## Discussion

Multiplexed NGS is currently essential for performing scRNA-seq in a cost-efficient manner. In order fully realize the advantage of the decreased costs associated with sequencing on platforms that utilize Illumina’s ExAMP chemistry, it is necessary for scRNA-seq libraries to utilize a multiplex sequencing strategy that adequately addresses the problem of index hopping. With the development of TruDrop, we take a preventative approach in utilizing a unique dual-indexing method that minimizes sample cross-talk [5]. Most prior work on high-throughput scRNA-seq libraries has focused on using computational methods to deconvolve and filter out entire barcodes (cells) with reads that could have originated from index-hopped sequencing reads, resulting in substantial data loss [8]. The V3 inDrop library structure has previously endeavored to implement a dual-indexed system for high-throughput scRNA-seq [7]. Its use of a portion of the cell barcode as the i7 index, however, means that the i7 index could be repeated across samples. It is thus a combinatorial dual-indexed system that does not resolve the cross-sample barcode collision problem. The work documented here allows for the independent evaluation of samples when filtering for barcode collisions, resulting in an increased retention of cell barcodes compared with that of single-indexed samples. Users who do not have access to the NovaSeq can also use this dual-indexed design for decreased cross-sample contamination on the HiSeq 3000, HiSeq 4000, and HiSeq X Ten, which also rely on patterned flow cells and ExAmp chemistry. Meanwhile, users who are restricted to sequencing inDrop libraries on the NextSeq platform, but still wish to use standard Illumina sequencing primers can use a single-indexed version via the universal TruSeq P5 (cell barcode + UMI) structure.

The structure used in this work enables sequencing compatibility with multiplexed pools of other Illumina compatible sequencing libraries unlike the prior inDrop V2 structure. We reason that the greatly reduced sequencing efficiency of the older V2 libraries alongside many other Illumina libraries on the NovaSeq is due to the use of custom sequencing primers. The V2 custom sequencing primers share a 13 bp sequence at the 5′ end of the i7 index primer with the Illumina TruSeq sequencing primers, allowing for the mis-priming of both inDrop and Illumina clusters on the flow cell. Sequencing pools with lower percentages of inDrop libraries likely result in greater opportunities for mis-priming events than sequencing a pool consisting entirely of inDrop libraries, as we have done on the NextSeq. This problem can likely be resolved by scaling of the ratio of custom primers used to standard primers, but this will proportionally reduce yield of standard Illumina libraries. Thus, our engineered TruDrop library structure provides the best solution by enabling complete compatibility between inDrop scRNA-seq libraries and standard Illumina libraries.

This potential to sequence TruDrop libraries alongside multiplexed pools of Illumina libraries enables much lower (3.7-fold decrease) base-calling error rates compared with libraries on the NextSeq without loss of sequencing read depth. This substantial improvement of sequencing quality is maintained when 24 TruDrop samples (30% of a run) were sequenced alongside Illumina libraries, with no effect on the quality of the standard libraries. The reduction in the base-calling error rate observed with the TruDrop on the NovaSeq is likely the major contributor to the increase in percent of uniquely aligned reads to the reference genome, as more accurate reads should result in a lower rate of ambiguous alignments. The uniquely aligned reads are those that move on to downstream data analysis, and thus, this improvement results in substantially more useable data. As for the discrepancy in the percentage of uniquely aligned reads between mouse (73%) and human (87%), this is a routinely observed difference between mapping to reference genomes of mouse versus human. Furthermore, the TruDrop libraries did not generate biased results, as sequencing the same samples using either library structures recovered the same cell types, with TruDrop libraries producing higher quality data.

## Conclusion

In summary, the TruDrop library structure resulted in the ability to sequence inDrop libraries on the NovaSeq by solving the problems of index hopping and library incompatibility. The resulting sequencing data have lower base call error rates, likely due to increased diversity of libraries sequenced from high multiplexity, resulting in better sequence alignments. The adoption of high-throughput next generation sequencing technologies results in substantial cost savings that should enable large scale cohort studies, with hundreds of samples, to be assayed by scRNA-seq.

## Methods

### Rationale of Library and primer design

The standard Illumina TruSeq library incorporates the following adapter sequences on either end of the library respectively:

P7: 5′ – CAAGCAGAAGACGGCATACGAGAT[i7]GTGACTGGAGTTCAGACGTGTGCTCTTCCGATCT – 3′.

P5: 5′ – AATGATACGGCGACCACCGAGATCTACAC[i5]ACACTCTTTCCCTACACGACGCTCTTCCGATCT – 3′.

The sequence present on the 5′ side of the i7 and i5 indexes are the adapter sequence required for annealing and cluster formation on the Illumina flow cell. The sequences to the 3′ side of the i7 and i5 indexes are where the TruSeq sequencing primers will bind during the sequencing process.

The sequence of the inDrop V2 library structure is as follows:

Cell Barcode + UMI(P7): 5′ – CAAGCAGAAGACGGCATACGAGAT [i7] CTCTTTCCCTACACGACGCTCTTCCGATCT [cell barcode 1] GAGTGATTGCTTGTGACGCCTT [Cell barcode 2] [UMI] TTTTTTTTTTTTTTTTTTT… – 3′.

Transcript (P5): 5′ – AATGATACGGCGACCACCGAGATCTACACGGTCTCGGCATTCCTGCTGAACCGCTCTTCCGATCTNNNNNN… – 3′.

For the cell barcode + UMI side of the V2 library structure, a truncated version of the Illumina i5 sequencing primer site was used as the sequencing primer for the cell barcode + UMI (P7 side). On the P5 - transcript side of the inDrop V2 library, a sequencing primer site that is currently considered obsolete by Illumina was used. This obsolete priming site on the P5 side of the V2 structure is added on via the use of a random hexamer during the 2nd RT and is then extended to the complete P5 V2 structure during a brief PCR. The truncated P5 sequencing priming site used on the P7 side of the V2 library is partly built into the primer sequence attached to the hydrogel bead used to capture the transcriptomic material during encapsulation. This truncated Illumina P5 primer sequence used on the P7 side has 13 bases in common with the full-length standard Illumina P7 primer sequenced. This will likely result in mis-priming events on inDrop libraries when sequencing inDrop V2 alongside large numbers of Illumina libraries. As a result, as the percentage of the library pool that is made up of Illumina libraries increases, the number of inDrop library strands that mis-primes during the i7 index read will also increase, resulting in progressive data loss. Theoretically, the index read for the sequencing clusters that can be properly identified will be of lower quality. The P5 side of the V2 structure could be changed due to its priming with a random hexamer. The P7 side could be changed so long as the resulting structure used the Illumina P5 sequencing primer site present on the primer used by the V2 hydrogel beads.

For the new TruSeq-inDrop (TruDrop) library structure the P7 and P5 sides were swapped so that the sequencing primer and flow cell binding site for the cell barcode + UMI side of the library followed Illumina’s TruSeq libraries. This was to prevent sequencing mis-priming events. The transcript side of the library now uses the P7 structure of TruSeq [14, 15]. The sequence for the final TruDrop library is as follows:

Transcript (P7): 5′ – CAAGCAGAAGACGGCATACGAGAT [i7] GTGACTGGAGTTCAGACGTGTGCTCTTCCGATCTNNNNNN… – 3′.

Cell Barcode + UMI (P5): 5′ – AATGATACGGCGACCACCGAGATCTACAC [i5] ACACTCTTTCCCTACACGACGCTCTTCCGATCT [cell barcode 1] GAGTGATTGCTTGTGACGCCTT [cell barcode 2] [UMI] TTTTTTTTTTTTTTTTTTT… -3′.

The new TruDrop library structure utilizes the standard Illumina TruSeq sequencing primers. It also incorporates a unique i7 and unique i5 index for each sample to address the previously-documented problem of index-hopping. The i7 and i5 index pairs were picked from the set of 96 pairs of unique dual indexes that Illumina has published as the “IDT for Illumina TruSeq UD Indexes”. These indexes have an edit distance of at least 2 for all i7 and for all i5. The TruDrop library preparation follows the same steps as previously published for the V2 library with the substitution of the following primers for their V2 counterparts:

TruDrop 2nd RT primer: 5′ – GTGACTGGAGTTCAGACGTGTGCTCTTCCGATCTNNNNNN – 3′.

TruDrop PE1: 5′ – AATGATACGGCGACCACCGAGATCTACAC [i5] ACACTCTTTCCCTACACGA – 3′.

TruDrop PE2: 5′ – CAAGCAGAAGACGGCATACGAGAT [i7] GTGACTGGAGTTCAGACGTGT – 3′.

TruDrop 2nd RT primer was ordered from IDT as desalted. TruDrop PE1 and PE2 primers were all ordered from IDT as TruGrade HPLC purified primers in individual tubes to minimize risk of cross-contamination during synthesis and handling. V2 PE2-N6 primer was ordered as desalted from Sigma. V2 PE1 and PE2 primers were ordered PAGE purified from Sigma. Primers were all resuspended at 100 μM in 10 mM Tris-HCl pH 8.0 and 0.1 mM EDTA pH 8.0. PE1 and PE2 primers were then diluted to 10 μM. For V2 libraries PE1 was mixed with PE2 in a 1:1 ratio (concentration of 5 μM for each primer) for working aliquots. For TruDrop libraries, unique dual-index primer pairs were then mixed in 1:1 ratio (concentration of 5 μM for each primer) for working aliquots.

### Calculation of cross-sample barcode collision as a result of index hopping

An estimate of the number of barcodes/cells to be discarded per sample can be calculated as follows. A prior study [5] documents the index hopping rate on a NovaSeq run to be 4.85%. Assuming it is equally likely for any given read to hop from one sample to the next, all of the samples should be treated as if all of the cells that they contain belong to a single sample. The manner of calculating rates of barcode collision for inDrop libraries was previously documented by [1, 2, 17, 18]. Rates of barcode collision for pools of 2, 4, 12, 24, and 48 samples (6000, 12,000, 36,000, 72,000, and 144,000 cells. respectively). Barcode collision and index hopping are 2 independent events so the probability of either occurring in a set number of cells is *P*(*barcode collision*) + *P*(*index hop*) − *P*(*barcode collision and index hop*). The resulting rate represents the percentage of cell barcodes discarded due to cross-sample barcode collision.

### Mouse colonic crypt isolation and dissociation

*Lrig1*^*CreERT2*^ and *Apc*^*fl*^ mice on C57BL/6 background were purchased from Jackson Laboratory. At 12 weeks, mice received 1–3 colonoscopy guided orthotropic injections of 0.70 mL of 100 μM 4-hydroxytamoxifen [26]. The following day, mice were administered 2.5% DSS (TdB consultancy, batch DB001–37) in deionized water for 6 days in their drinking water. Mice were sacrificed 28 days after 4-hydroxytamoxifen injections via CO_2_ asphyxiation and cervical dislocation. Colonic tissues were dissected and incubated in chelation buffer (3 mM EDTA, 0.5 mM DTT) at 4 °C for 1 h and 15 min. Tissues were shaken in 10 mL of PBS in a 15 mL conical tube for 2 min to release the crypts. The crypt suspension was centrifuged at 250–300 xg for 5 min at 4 °C. Crypts were washed three times with 1x DPBS. The crypts were dissociated into single cells using a cold-activated protease (1 mg/mL) and DNase I (2.5 mg/mL) mixture in 1x DPBS on a rocker at 4 °C for 25 min. Single cells were then washed three times with 1x DPBS after spinning 600x g for 5 min each at 4 °C.

### Human colonic crypt isolation and dissociation

Colonic biopsies were collected and placed into RPMI or UW (University of Wisconsin) solution prior to processing. Upon arrival, biopsies were minced to 4 mm^2^ and washed with 1x DPBS. They were then incubated in chelation buffer (4 mM EDTA, 0.5 mM DTT) at 4 °C for 1 h 15 min. Tissues were then dissociated with cold protease and DNase I for 25 min. Single-cell suspensions were triturated at the start and every 10 min with a P1000 pipette tip with the tip 0.1–0.5 cm removed. Single cells were washed three times with 1x DPBS after spinning 600 xg for 5 min each at 4 °C.

### inDrop single-cell encapsulation and Library preparation

A target of 3000 single cells per sample were encapsulated and barcoded using the inDrop platform with 1Cell-Bio library preparation protocol version 2.3. Modifications to the protocol include reverse transcription as noted in [27], ExoI digestion, second strand synthesis, and T7 in vitro transcription as noted in version 1.2. Furthermore, the volumes of diagnostic qPCR and final PCR steps were doubled, with a final double-sized size selection. For TruDrop-specific modifications, TruDrop custom primers were used (RT, PE1, PE2).

### TruDrop primer testing via qPCR

To test the efficiency of TruDrop dual indexing primers, a single mouse inDrop library was prepared up through the second RT using the TruDrop RT primer. The sample was used to run a diagnostic qPCR for each pair of TruDrop i7 and i5 indexes, all in parallel, on a BioRad C1000 Touch Thermal Cycler CFX96 Real-time system. To verify that the TruDrop primers amplified appropriately, their amplification curves were compared with two V2 libraries that had previously produced good results on the NextSeq. An index pair not reaching the Ct value of 5000 RFU was not included in subsequent analysis. Based off of prior testing by [28], it was expected that the Ct for individual primer pairs would not deviate from the average by more than 1.5 cycles.

### Illumina Sequencing

All libraries were evaluated on a Qubit 3.0 fluorometer and an Agilent 2100 Bioanalyzer regarding concentration and fragment size distribution prior to sequencing on various platforms.

#### NextSeq

V2 libraries were sequenced on the NextSeq 500 using a PE 75 kit in a customized sequencing run as previously done [24]. 10–15% PhiX was pooled when appropriate with the resulting runs also using some sequencing cycles to read the i5 sequence of the PhiX to easily distinguish it from the V2 libraries during sample demultiplexing.

#### MiSeq

Sequencing of a V2 library on the MiSeq was performed using the Reagent Kit v2 Nano with custom sequencing primers, along with a 10% PhiX spike-in. Sequencing was performed using 30 cycles for read 1 (transcript), 6 cycles for the index read, and 30 cycles for read 2 (cell barcode + UMI).

#### iSeq 100

TruDrop libraries were sequenced on the iSeq with a 10% PhiX spike-in using a PE 150 kit. The cell barcode + UMI was sequenced on read 1. The transcript was sequenced on read 2.

#### NovaSeq 6000

Sequencing on the NovaSeq was performed using a S4 flow cell with a PE 150 kit. TruDrop libraries, at a 2 nM standard loading concentration, were pooled with other Illumina compatible libraries, and sequenced to various target depths (50–500 million reads).

### Downstream data analysis

For all sequence data, reads were demultiplexed using bcl2fastq v2.20.0.422. Base call accuracy (% > = Q30 score) plots were generated via Illumina’ BaseSpace. Quality scores were generated using fastQC to find the average quality score per cycle for reads from the demultiplexed fastq files [16]. The proportion for how much each cycle was contributing to each transcript, barcode 1, barcode 2, and UMI read was determined and used to calculate the weighted average of the quality score for the transcript (first 100 bases only) and cell barcodes + UMI. Base call error rates were then calculated using the formula *p* = 10^(−*Q*/10)^. The means of the transcript and cell barcodes + UMI were used to perform Mann-Whitney tests.

Following demultiplexing, reads were filtered, sorted by their barcode of origin, and aligned to the reference transcriptome to generate a counts matrix using the DropEst pipeline [29]. Barcodes containing cells were filtered for further analysis, as previous [23], and aligned using Harmony [30]. t-SNE and sc-UniFrac analyses were performed following previous methods [23, 25] in Matlab (Mathworks) and R, respectively.

## Supplementary information

**Additional file 1: Supplementary Figure 1.** Comparison of amplification of TruDrop and inDrop V2 primers during library preparation. (A) Diagnostic qPCR amplification curves comparing performance of all TruDrop primer pairs to V2 primers, all performed on the same sample. (B) Ct values of A. **Supplementary Figure 2.** Comparison of the library size distributions for TruDrop and inDrop V2 structured libraries during library preparation. (A) A BioAnalyzer profile of the size distribution of a V2 structured library. The spikes at 35 bp and 10,380 bp are controls. The numbers above the profile indicate the timepoints at which the various peaks were measured. (B) A BioAnalyzer profile of the size distribution of a TruDrop structured library. The spikes at 35 bp and 10,380 bp are controls. The numbers above the profile indicate the timepoints at which the various peaks were measured. (C) Plot of the average library size as determined via a BioAnalyzer for inDrop V2 libraries and TruDrop libraries. The median value is marked with a dotted line and a 95% confidence interval for the median is shown. **Supplementary Figure 3.** Comparison of sequence alignment metrics of inDrop V2 on NextSeq and TruDrop on NovaSeq. (A) Plot of the percent of reads with Valid Barcodes in 11 inDrop V2 mouse libraries and 23 TruDrop mouse libraries. The Median value is marked with a dotted line and a 95% confidence interval for the median is shown. (B) Plot of the percent of reads that uniquely align to a section of the mouse genome for inDrop V2 libraries and TruDrop libraries. The median value is marked with a dotted line and a 95% confidence interval for the median is shown. (C) Plot of the percent of reads that contain valid cell barcodes and a transcript that uniquely aligns to a section of the mouse genome for inDrop V2 libraries and TruDrop libraries. The median value is marked with a dotted line and a 95% confidence interval for the median is shown. **Supplementary Figure 4.** Another comparison of cell types identified between inDrop V2 on NextSeq and TruDrop on NovaSeq. (A) t-SNE and (B) sc-UniFrac analysis as performed in Fig. 5.

**Additional file 2: Supplementary Table 1**. Cost of Sequencing for inDrop. **Supplementary Table 2**. Evaluation of two TruDrop libraries’ raw yield and quality in low-throughput sequencing run on the iSeq 100. **Supplementary Table 3**. 24 TruDrop libraries raw data yield and quality in combined high-throughput sequencing run on the NovaSeq. **Supplementary Table 4**. 37 inDrop library quality scores from TruDrop on NovaSeq and V2 on NextSeq. **Supplementary Table 5**. inDrop library alignment metrics from TruDrop on NovaSeq and V2 on NextSeq. **Supplementary Table 6**. Diversity of UMI’s and genes expressed for cells sequenced with the TruDrop structure.

**Additional file 3: Supplementary file 1**.

**Additional file 4: Supplementary file 2**.

**Additional file 5: Supplementary file 3**.

## Data Availability

The datasets analyzed during the current study are not publicly available due to them being part of the Human Tumor Atlas Network, and will be made publicly available through the data coordinating center. These data are also available from the corresponding author on reasonable request.

## Abbreviations

scRNA-seq: Single-cell RNA sequencing
ExAmp: Exclusion Amplification
NGS: Next Generation Sequencing
V2: Version of inDrop built from the second iteration of the gel beads
dsDNA: Double-stranded DNA
TruDrop: TruSeq-inDrop library structure that builds upon the inDrop V2 beads
i7: Illumina’s acronym for the first index
i5: Illumina’s acronym for the second index
PhiX: a control library with a diverse base composition sourced from Illumina
Q30: Phred scaled sequencing quality score of 30
UMI: Unique molecular identifier
P5: Flow cell binding site on the side of the i5 index for Illumina libraries
P7: Flow cell binding site on the side of the i7 index for Illumina libraries
PCR: Polymerase chain reaction
qPCR: Quantitative polymerase chain reaction
