# Supplementary figures and images for: Dual indexed library design enables compatibility of in-Drop single-cell RNA-sequencing with exAMP chemistry sequencing platforms
Source: BMC Genomics. 2020 Jul 2;21:456. doi: 10.1186/s12864-020-06843-0 (PMC7331155; doi:10.1186/s12864-020-06843-0)

A

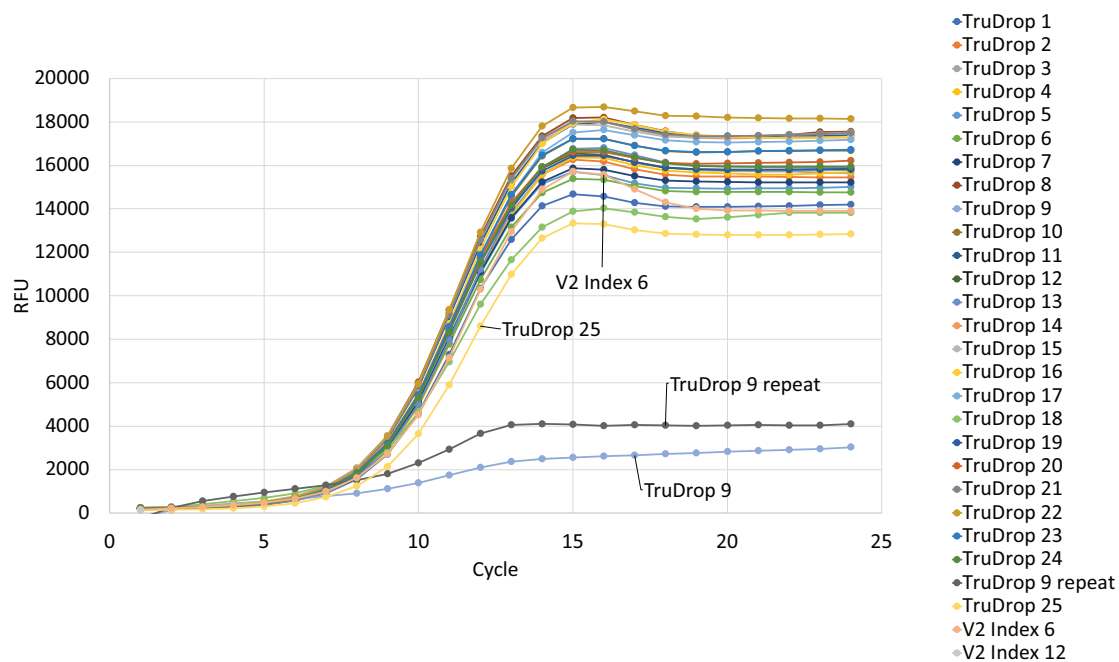

B

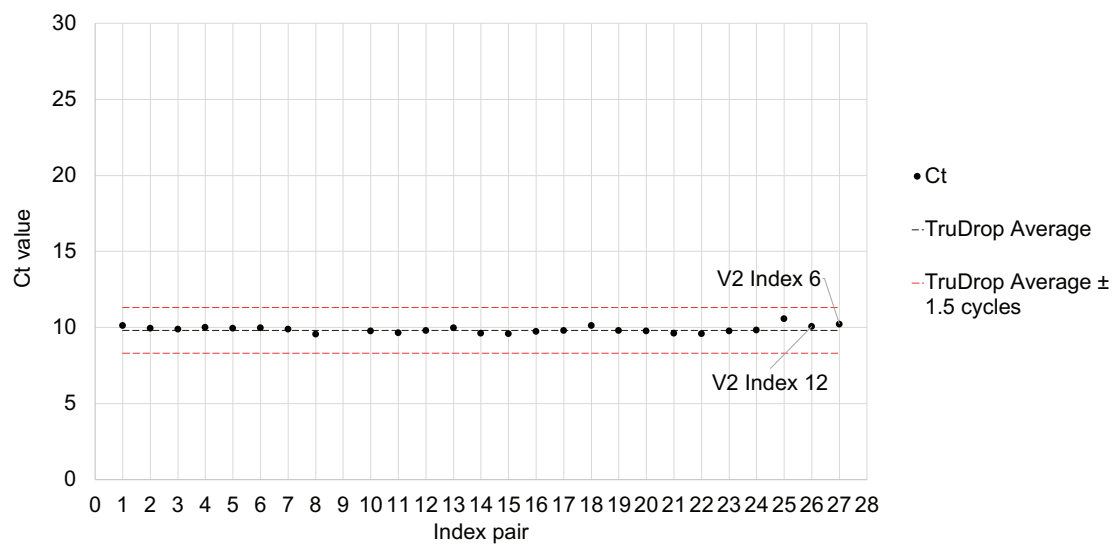

Supplementary Figure 1

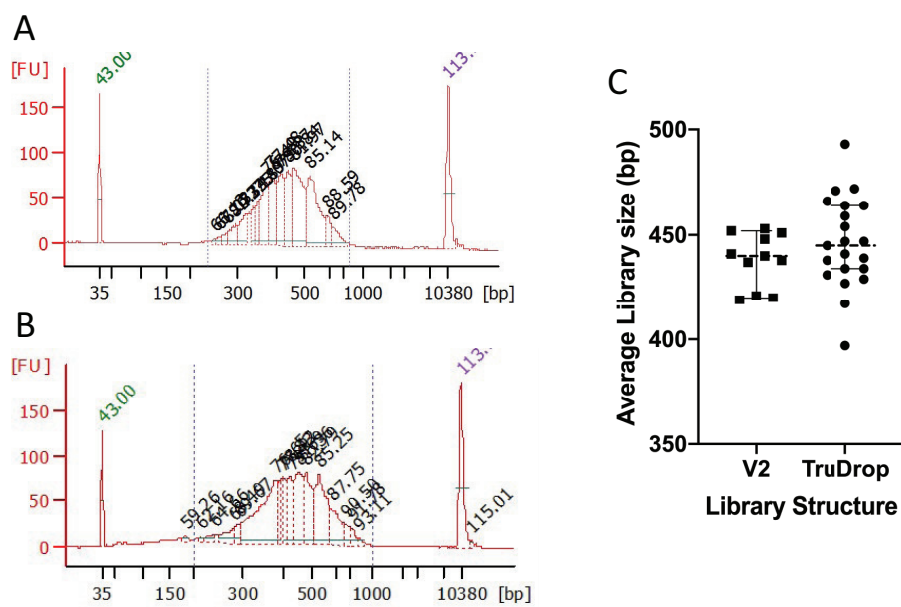

Supplementary Figure 2

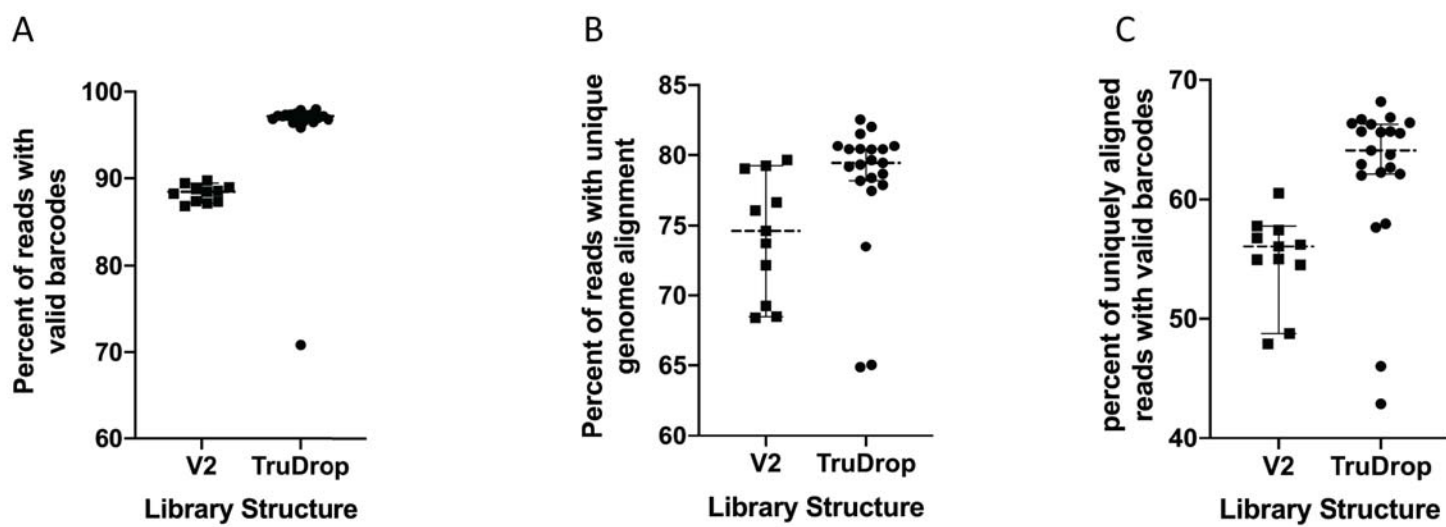

Supplementary Figure 3

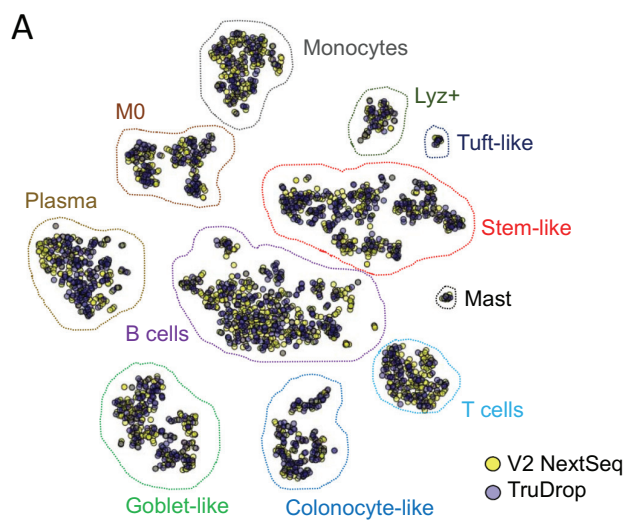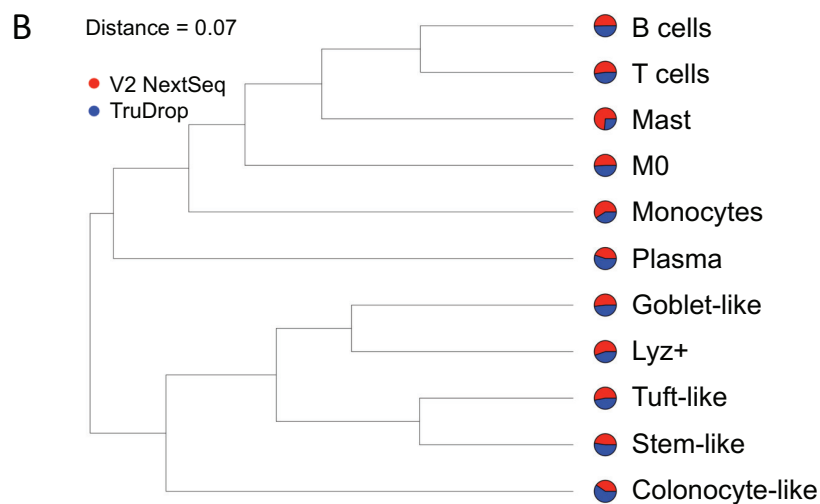

**Supplementary Figure 4**

Supplement: Supplementary file 1 — Additional file 1: Supplementary Figure 1. Comparison of amplification of TruDrop and inDrop V2 primers during library preparation. (A) Diagnostic qPCR amplification curves comparing performance of all TruDrop primer pairs to V2 primers, all performed on the same sample. (B) Ct values of A. Supplementary Figure 2. Comparison of the library size distributions for TruDrop and inDrop V2 structured libraries during library preparation. (A) A BioAnalyzer profile of the size distribution of a V2 structured library. The spikes at 35 bp and 10,380 bp are controls. The numbers above the profile indicate the timepoints at which the various peaks were measured. (B) A BioAnalyzer profile of the size distribution of a TruDrop structured library. The spikes at 35 bp and 10,380 bp are controls. The numbers above the profile indicate the timepoints at which the various peaks were measured. (C) Plot of the average library size as determined via a BioAnalyzer for inDrop V2 libraries and TruDrop libraries. The median value is marked with a dotted line and a 95% confidence interval for the median is shown. Supplementary Figure 3. Comparison of sequence alignment metrics of inDrop V2 on NextSeq and TruDrop on NovaSeq. (A) Plot of the percent of reads with Valid Barcodes in 11 inDrop V2 mouse libraries and 23 TruDrop mouse libraries. The Median value is marked with a dotted line and a 95% confidence interval for the median is shown. (B) Plot of the percent of reads that uniquely align to a section of the mouse genome for inDrop V2 libraries and TruDrop libraries. The median value is marked with a dotted line and a 95% confidence interval for the median is shown. (C) Plot of the percent of reads that contain valid cell barcodes and a transcript that uniquely aligns to a section of the mouse genome for inDrop V2 libraries and TruDrop libraries. The median value is marked with a dotted line and a 95% confidence interval for the median is shown. Supplementary Figure 4. Another comparison of [file 12864_2020_6843_MOESM1_ESM.pdf]

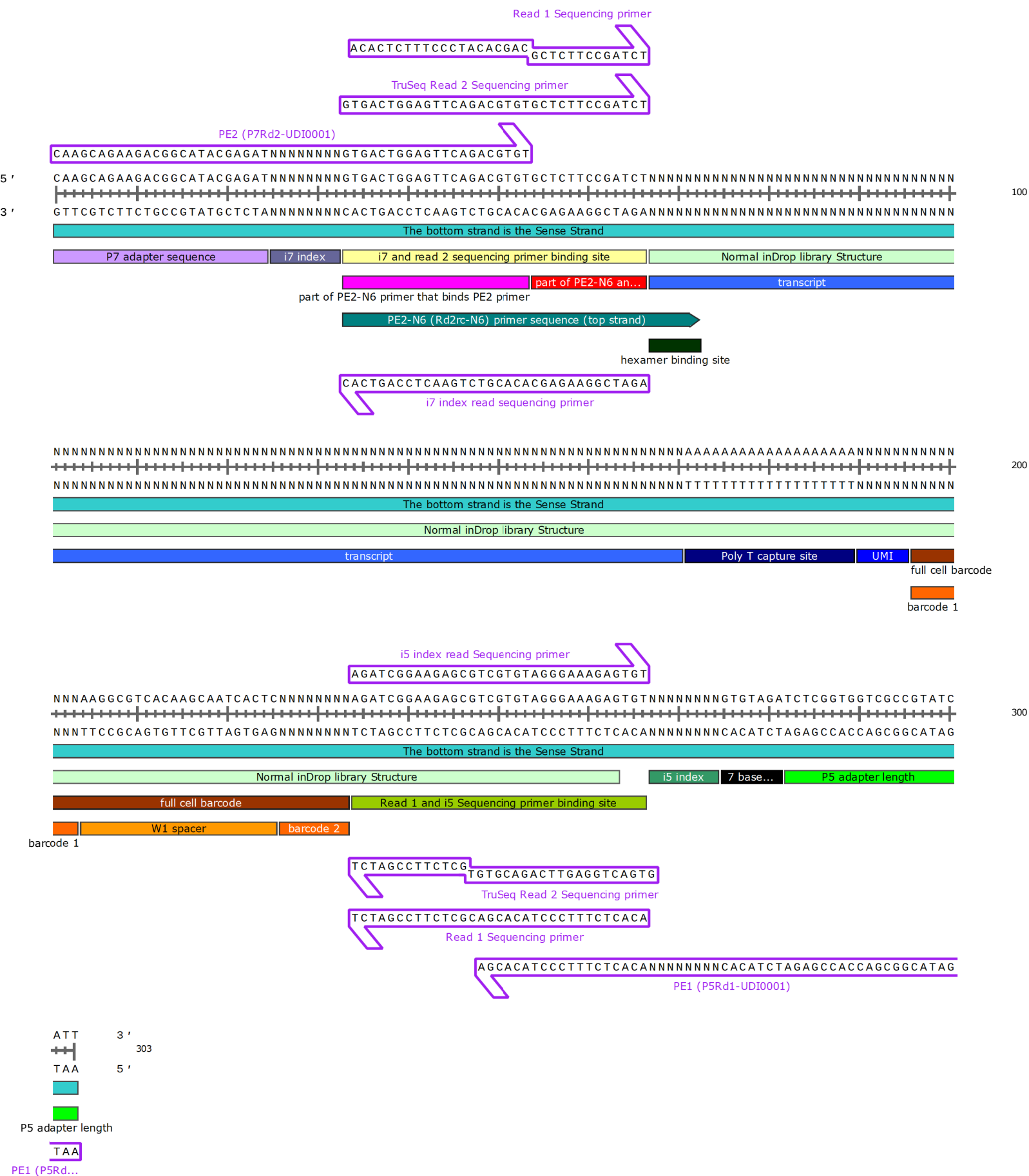

Supplement: Supplementary file 5 — Additional file 5: Supplementary file 3. [file 12864_2020_6843_MOESM5_ESM.pdf]
