# Supplementary material for: Dual indexed library design enables compatibility of in-Drop single-cell RNA-sequencing with exAMP chemistry sequencing platforms
Source: BMC Genomics. 2020 Jul 2;21:456. doi: 10.1186/s12864-020-06843-0 (PMC7331155; doi:10.1186/s12864-020-06843-0)
Supplement: Supplementary file 2 — Additional file 2: Supplementary Table 1. Cost of Sequencing for inDrop. Supplementary Table 2. Evaluation of two TruDrop libraries’ raw yield and quality in low-throughput sequencing run on the iSeq 100. Supplementary Table 3. 24 TruDrop libraries raw data yield and quality in combined high-throughput sequencing run on the NovaSeq. Supplementary Table 4. 37 inDrop library quality scores from TruDrop on NovaSeq and V2 on NextSeq. Supplementary Table 5. inDrop library alignment metrics from TruDrop on NovaSeq and V2 on NextSeq. Supplementary Table 6. Diversity of UMI’s and genes expressed for cells sequenced with the TruDrop structure. [file 12864_2020_6843_MOESM2_ESM.docx]

**Supplementary Table 1**. **Cost of Sequencing for inDrop^†^**

| Sequencer | Flow cell | Sequencing Kit | Cost of Flow cell | Number of lanes | Possible Number of Samples per flow cell | Sequencing Cost per sample |
| --- | --- | --- | --- | --- | --- | --- |
| NextSeq | High Throughput | PE 75 | $3055.00 | 4 | 4 | $764.75 |
| NovaSeq | S2** | PE 150 | $9,840.00** | 2 | 37 | $531.89 |
| NovaSeq | S4 | PE 150 | $36,135.00 | 4 | 96* | $361.35 |

^†^assuming a read dept of 100 million reads (30 billion bases) per sample and unless otherwise noted costs are from local sequencing core facility

*Assumes sharing flow cell with other users

** requires lane splitting and cost are from University of Wisconsin (<https://www.biotech.wisc.edu/services/dnaseq/pricing>)

**Supplementary Table 2. Evaluation of two TruDrop libraries’ raw yield and quality in low-throughput sequencing run on the iSeq 100**

| Library | Sequencer | i7 | I5 | Expected Reads | Observed inDrop Reads* |
| --- | --- | --- | --- | --- | --- |
| Mouse 4 | iSeq 100 | CCGCGGTT | AGCGCTAG | 2,000,000 | 2,876,464 |
| Mouse 5 | iSeq 100 | TTATAACC | GATATCGA | 2,000,000 | 3,166,938 |

*Libraries were sequenced alongside a 10% spike-in of PhiX.

The TruSeq-inDrop (TruDrop) libraries using both an i7 and i5 index saw about 1.5x the expected yield on the iSeq sequencer indicating that for this test the flow cell over-clustered. The iSeq uses similar chemistry to that of the NovaSeq. This shows that the TruDrop structured libraries could be sequenced on the NovaSeq.

**Supplementary Table 3. 24 TruDrop libraries raw data yield and quality in combined high-throughput sequencing run on the NovaSeq**

| Sample | i7 | i5 | Expected inDrop Reads | Observed inDrop reads | Average % of the lane | perfect index read (%) | Mean Barcodes and UMI Quality Score | Mean Transcript Quality Scores |
| --- | --- | --- | --- | --- | --- | --- | --- | --- |
| Human 1 | CCGCGGTT | AGCGCTAG | 125,000,000 | 163,510,352 | 1.38 | 96.84 | 36.05 | 35.36 |
| Human 2 | TTATAACC | GATATCGA | 125,000,000 | 141,764,120 | 1.20 | 96.44 | 36.02 | 35.34 |
| Human 3 | GGACTTGG | CGCAGACG | 125,000,000 | 127,912,777 | 1.08 | 96.91 | 35.97 | 35.44 |
| Human 4 | AAGTCCAA | TATGAGTA | 125,000,000 | 141,900,719 | 1.20 | 96.83 | 36.03 | 35.41 |
| Human 5 | ATCCACTG | AGGTGCGT | 125,000,000 | 153,271,668 | 1.29 | 97.04 | 36.02 | 35.36 |
| Human 6 | TTGGACTC | GGAAGCAG | 125,000,000 | 143,214,632 | 1.21 | 97.09 | 36.08 | 35.46 |
| Human 7 | GGCTTAAG | TCGTGACC | 125,000,000 | 121,001,482 | 1.03 | 96.30 | 36.09 | 35.34 |
| Human 8 | AATCCGGA | CTACAGTT | 125,000,000 | 117,718,028 | 1.00 | 96.35 | 36.10 | 34.59 |
| Human 9 | TAATACAG | ATATTCAC | 125,000,000 | 176,705,278 | 1.50 | 96.49 | 36.01 | 35.30 |
| Human 10 | CGGCGTGA | GCGCCTGT | 125,000,000 | 176,054,943 | 1.51 | 95.49 | 36.04 | 35.37 |
| Human 11 | ATGTAAGT | ACTCTATG | 125,000,000 | 164,005,038 | 1.38 | 97.05 | 36.11 | 35.20 |
| Human 12 | GCACGGAC | GTCTCGCA | 125,000,000 | 150,680,775 | 1.26 | 97.57 | 36.04 | 35.31 |
| Human 13 | GGTACCTT | AAGACGTC | 125,000,000 | 170,171,924 | 1.43 | 97.14 | 36.09 | 35.29 |
| Human 14 | AACGTTCC | GGAGTACT | 125,000,000 | 128,785,547 | 1.09 | 96.74 | 36.12 | 35.36 |
| Human 15 | ACTAAGAT | AACCGCGG | 125,000,000 | 150,500,764 | 1.31 | 93.28 | 36.14 | 35.18 |
| Mouse 6 | GCTTGTCA | GAACATAC | 125,000,000 | 131,683,586 | 1.11 | 96.71 | 36.09 | 35.16 |
| Mouse 7 | CAAGCTAG | ACATAGCG | 125,000,000 | 168,538,426 | 1.42 | 96.84 | 36.10 | 35.43 |
| Mouse 8 | TGGATCGA | GTGCGATA | 125,000,000 | 124,903,031 | 1.05 | 97.61 | 36.04 | 35.42 |
| Mouse 9 | GACCTGAA | TTGGTGAG | 125,000,000 | 125,928,312 | 1.08 | 95.63 | 36.07 | 35.24 |
| Mouse 10 | TCTCTACT | CGCGGTTC | 125,000,000 | 132,604,827 | 1.13 | 96.32 | 36.10 | 35.31 |
| Mouse 11 | CTCTCGTC | TATAACCT | 125,000,000 | 121,444,989 | 1.03 | 96.79 | 36.16 | 35.53 |
| Mouse 12 | CCAAGTCT | AAGGATGA | 125,000,000 | 127,434,526 | 1.08 | 96.80 | 36.12 | 35.48 |
| Mouse 13 | GCAGAATT | ACCGGCCA | 125,000,000 | 188,900,350 | 1.61 | 95.62 | 36.11 | 35.45 |
| Mouse 14 | ATGAGGCC | GTTAATTG | 125,000,000 | 126,939,171 | 1.08 | 96.08 | 36.06 | 35.30 |

**Supplementary Table 4. 37 inDrop library quality scores from TruDrop on NovaSeq and V2 on NextSeq**

| Sample | Library Structure | Sequencer | Mean Barcodes and UMI Quality Score | Mean Transcript Quality Scores |
| --- | --- | --- | --- | --- |
| Human 1 | TruDrop | NovaSeq | 36.05 | 35.36 |
| Human 2 | TruDrop | NovaSeq | 36.02 | 35.34 |
| Human 3 | TruDrop | NovaSeq | 35.97 | 35.44 |
| Human 4 | TruDrop | NovaSeq | 36.03 | 35.41 |
| Human 5 | TruDrop | NovaSeq | 36.02 | 35.36 |
| Human 6 | TruDrop | NovaSeq | 36.08 | 35.46 |
| Human 7 | TruDrop | NovaSeq | 36.09 | 35.34 |
| Human 8 | TruDrop | NovaSeq | 36.10 | 34.59 |
| Human 9 | TruDrop | NovaSeq | 36.01 | 35.30 |
| Human 10 | TruDrop | NovaSeq | 36.04 | 35.37 |
| Human 11 | TruDrop | NovaSeq | 36.11 | 35.20 |
| Human 12 | TruDrop | NovaSeq | 36.04 | 35.31 |
| Human 13 | TruDrop | NovaSeq | 36.09 | 35.29 |
| Human 14 | TruDrop | NovaSeq | 36.12 | 35.36 |
| Human 15 | TruDrop | NovaSeq | 36.14 | 35.18 |
| Mouse 4 | TruDrop | NovaSeq | 36.22 | 35.57 |
| Mouse 5 | TruDrop | NovaSeq | 36.19 | 35.53 |
| Mouse 6 | TruDrop | NovaSeq | 36.09 | 35.16 |
| Mouse 7 | TruDrop | NovaSeq | 36.10 | 35.43 |
| Mouse 8 | TruDrop | NovaSeq | 36.04 | 35.42 |
| Mouse 9 | TruDrop | NovaSeq | 36.07 | 35.24 |
| Mouse 10 | TruDrop | NovaSeq | 36.10 | 35.31 |
| Mouse 11 | TruDrop | NovaSeq | 36.16 | 35.53 |
| Mouse 12 | TruDrop | NovaSeq | 36.12 | 35.48 |
| Mouse 13 | TruDrop | NovaSeq | 36.11 | 35.45 |
| Mouse 14 | TruDrop | NovaSeq | 36.06 | 35.30 |
| Mouse 15 | V2 | NextSeq | 30.73 | 30.55 |
| Mouse 16 | V2 | NextSeq | 32.56 | 29.88 |
| Mouse 17 | V2 | NextSeq | 32.55 | 29.85 |
| Mouse 18 | V2 | NextSeq | 32.62 | 29.78 |
| Mouse 19 | V2 | NextSeq | 32.55 | 29.69 |
| Mouse 4 | V2 | NextSeq | 31.89 | 28.67 |
| Mouse 20 | V2 | NextSeq | 32.02 | 28.84 |
| Mouse 21 | V2 | NextSeq | 32.05 | 28.76 |
| Mouse 5 | V2 | NextSeq | 32.03 | 28.64 |
| Mouse 22 | V2 | NextSeq | 30.88 | 31.11 |
| Mouse 23 | V2 | NextSeq | 31.08 | 31.37 |

**Supplementary Table 5. inDrop library alignment metrics from TruDrop on NovaSeq and V2 on NextSeq**

| Sample | Library Structure | Sequencer | Percentage of reads with valid cell barcodes | Percentage of reads with unique alignment to genome | Percentage of reads with unique alignment to genome and valid cell barcodes |
| --- | --- | --- | --- | --- | --- |
| Mouse 22 | V2 | NextSeq | 88.4615931 | 68.50 | 48.7697457 |
| Mouse 23 | V2 | NextSeq | 88.2396626 | 68.41 | 47.8950666 |
| Mouse 15 | V2 | NextSeq | 89.7426661 | 79.05 | 56.2039767 |
| Mouse 16 | V2 | NextSeq | 89.4440407 | 79.25 | 60.5255184 |
| Mouse 17 | V2 | NextSeq | 88.5607802 | 76.65 | 54.4887237 |
| Mouse 18 | V2 | NextSeq | 88.9566015 | 79.66 | 57.4419159 |
| Mouse 19 | V2 | NextSeq | 88.7766165 | 74.63 | 54.9204296 |
| Mouse 4 | V2 | NextSeq | 87.128928 | 73.72 | 56.7835069 |
| Mouse 20 | V2 | NextSeq | 87.3496454 | 72.14 | 56.0598331 |
| Mouse 21 | V2 | NextSeq | 86.8311688 | 76.07 | 57.7888196 |
| Mouse 5 | V2 | NextSeq | 87.3907899 | 69.26 | 54.988921 |
| Mouse 4 | TruDrop | NovaSeq | 97.8908116 | 65.03 | 57.6593948 |
| Mouse 5 | TruDrop | NovaSeq | 97.9865474 | 73.48 | 62.2444588 |
| Mouse 24 | TruDrop | NovaSeq | 96.8940531 | 80.43 | 66.6999663 |
| Mouse 25 | TruDrop | NovaSeq | 96.9431688 | 80.44 | 66.3672602 |
| Mouse 26 | TruDrop | NovaSeq | 97.2156963 | 80.46 | 65.6827311 |
| Mouse 27 | TruDrop | NovaSeq | 97.2289472 | 80.65 | 65.5298834 |
| Mouse 28 | TruDrop | NovaSeq | 97.0045908 | 79.19 | 62.6562926 |
| Mouse 29 | TruDrop | NovaSeq | 97.2432761 | 78.19 | 62.1237561 |
| Mouse 30 | TruDrop | NovaSeq | 96.5207427 | 79.45 | 66.4236919 |
| Mouse 31 | TruDrop | NovaSeq | 97.3869489 | 80.42 | 66.8583941 |
| Mouse 32 | TruDrop | NovaSeq | 97.3063865 | 82.55 | 57.9644893 |
| Mouse 33 | TruDrop | NovaSeq | 70.8353322 | 77.89 | 42.8844967 |
| Mouse 34 | TruDrop | NovaSeq | 97.1824752 | 81.53 | 66.2871647 |
| Mouse 35 | TruDrop | NovaSeq | 96.8066726 | 78.66 | 62.008565 |
| Mouse 36 | TruDrop | NovaSeq | 95.87355 | 79.64 | 62.9289587 |
| Mouse 37 | TruDrop | NovaSeq | 96.5048999 | 82.04 | 68.1791529 |
| Mouse 38 | TruDrop | NovaSeq | 97.3924132 | 77.46 | 65.6304498 |
| Mouse 39 | TruDrop | NovaSeq | 97.1867987 | 78.38 | 65.6829815 |
| Mouse 40 | TruDrop | NovaSeq | 97.4905632 | 80.65 | 64.091757 |
| Mouse 41 | TruDrop | NovaSeq | 97.3512778 | 79.33 | 63.7343648 |
| Mouse 42 | TruDrop | NovaSeq | 96.4349893 | 64.87 | 46.0166066 |

**Supplementary Table 6. Diversity of UMI’s and genes expressed for cells sequenced with the TruDrop structure**

| Sample | Reads/cell encapsulated | Cells detected | Median UMI’s/Cell  (25^th^ percentile, 75^th^ percentile) | Median Genes/Cell  (25^th^ percentile, 75^th^ percentile) |
| --- | --- | --- | --- | --- |
| TruDrop Human 1 | 19398 | 1246 | 4902 (3162, 7074) | 1916 (1292, 2498) |
| TruDrop Human 2 | 39302 | 927 | 10332 (6178, 15303) | 2393 (1674, 3241) |
| TruDrop Human 3 | 61974 | 1381 | 11545 (8043, 15921) | 3208 (2382, 4207) |
| TruDrop Human 4 | 149535 | 2049 | 16141 (12034, 21397) | 3760 (2985, 4537) |
